# Comparative Safety and Efficacy Profile of a Novel Oil in Water Vaccine Adjuvant Comprising Vitamins A and E and a Catechin in Protective Anti-Influenza Immunity

**DOI:** 10.3390/nu9050516

**Published:** 2017-05-21

**Authors:** Sapna Patel, Yasser Faraj, Debra K. Duso, William W. Reiley, Erik A. Karlsson, Stacey Schultz-Cherry, Michael Vajdy

**Affiliations:** 1 EpitoGenesis, Inc., 933 Hartford Turnpike Vernon, Hartford, CT 06066, USA; spatel1486@gmail.com (S.P.); yasserfaraj92@gmail.com (Y.F.); 2 TICRO at Trudeau Institute, 154 Algonquin Ave, Saranac Lake, NY 12983, USA; dduso@trudeauinstitute.org (D.K.D.); wreiley@trudeauinstitute.org (W.W.R.); 3 Department of Infectious Diseases, St. Jude Children’s Research Hospital, 262 Danny Thomas Place, Memphis, TN 38105, USA; erik.karlsson@stjude.org (E.A.K.); Stacey.Schultz-Cherry@STJUDE.ORG (S.S.-C.)

**Keywords:** vaccine, adjuvant, natural, vitamin A, vitamin E, flavonoid, catechin, influenza, immunity

## Abstract

Non-replicating vaccines, such as those based on recombinant proteins, require adjuvants and delivery systems, which have thus far depended on mimicking pathogen danger signals and strong pro-inflammatory responses. In search of a safer and more efficacious alternative, we tested whether vaccinations with influenza recombinant hemagglutinin (HA) mixed with a novel vegetable oil in water emulsion adjuvant (Natural Immune-enhancing Delivery System, NIDS), based on the immune-enhancing synergy of vitamins A and E and a catechin, could protect against intra-nasal challenge with live influenza virus. Vaccinations of inbred Brag Albino strain c (BALB/c) mice, with HA mixed with NIDS compared to other adjuvants, i.e., a squalene oil in water emulsion (Sq. oil), and the Toll Like Receptor 3 (TLR3) agonist Poly (I:C), induced significantly lower select innate pro-inflammatory responses in serum, but induced significantly higher adaptive antibody and splenic T Helper 1 (TH1) or TH2, but not TH17, responses. Vaccinations with NIDS protected against infection, as measured by clinical scores, lung viral loads, and serum hemagglutination inhibition titers. The NIDS exhibited a strong dose sparing effect and the adjuvant action of NIDS was intact in the outbred CD1 mice. Importantly, vaccinations with the Sq. oil, but not NIDS, induced a significantly higher Serum Amyloid P component, an acute phase reactant secreted by hepatocytes, and total serum IgE. Thus, the NIDS may be used as a clinically safer and more efficacious vaccine adjuvant against influenza, and potentially other infectious diseases.

## 1. Introduction

Most non-replicating vaccines require adjuvants and delivery systems. Several adjuvants and delivery systems for protein-, DNA-, and RNA-based vaccines have been devised and used in animal and early clinical studies [1]. Commercially available injectable inactivated whole- and split-virus influenza vaccines have been successful at preventing diseases caused by seasonal influenza infection [2,3], and a licensed cold adapted live attenuated influenza vaccine, administered intra-nasally, is currently in use [4]. However, the non-replicating vaccines suffer from limited efficacy in generating long-lasting immunity, particularly in the elderly and they are not sufficiently cross-reactive to protect against antigenic variants [5,6,7]. Thus, safe and effective vaccine adjuvants and delivery systems with the potential to simultaneously protect against multiple strains and clades of seasonal and pandemic influenza viruses are highly desirable.

In search of safer and more efficacious vaccine adjuvants and delivery systems, we focused on select vitamins and flavonoids. We previously demonstrated the ability of NIDS to enhance humoral and cellular immune responses against co-administered HIV-1 envelope antigens [8,9]. We have introduced and discussed the immunomodulating properties of vitamins A and E and flavonoids, including catechins and tannins, as the major components of NIDS, in two published reviews [10,11]. Flavonoids are a group of polyphenols found in fruits, vegetables, and green tea, and have been reported to demonstrate their benefits in lowering oxidative stress and also exert their beneficial effects on cardiovascular and chronic inflammatory diseases [10,11] Vitamin A (Vit A) is a fat-soluble vitamin that is derived from two sources: preformed retinoids and provitamin carotenoids. Retinoic acid (RA) and its parent compound retinol (ROH, vitamin A) have been used as important immunopotentiating agents since the early 1900s. Although vitamin E (Vit E), has been a component of a licensed influenza vaccine for humans, there have been many contradictory scientific reports on whether Vit E supplementation can improve immune responses in part by altering cytokine production [10,11]. We have designed a novel Nutritive Immune-enhancing Delivery System (NIDS), which is based on the synergistic immune enhancing properties of Vit A and/or Vit E and a catechin (epigallo catechin gallate; EGCG) in a vegetable oil (mustard seed) as an oil in water emulsion.

For prophylactic vaccines, the safety bar is rightfully set high, as the vaccinees are otherwise healthy with regards to the disease they are vaccinated against. Therefore, it is important to find biomarkers that define safety in preclinical studies. Serum Acute Phase Reactants (APR) could serve assuch safety biomarkers. APR refers to a group of proteins that change their concentration significantly in response to an inflammatory response such as an infection or a wound [12]. One readily measurable APR in serum is the Serum Amyloid P component (SAP) which is secreted by hepatocytes [13,14]. Another useful biosafety marker is serum total IgE, the relevance of which is highlighted by the findings that egg-based influenza vaccines cause vaccine-specific IgE responses [15]. Hence in this study we measured SAP and IgE in serum as a measure of vaccine safety.

In the present study, we compared the adjuvanticity of NIDS with that of two licensed adjuvants and delivery systems, i.e., aluminum hydroxide and the squalene oil based oil in water emulsion, which are known to preferentially induce T Helper 2 (TH2)-type responses [16]. As a control to preferentially induce TH1-type responses, we selected the Toll Like Receptor 3 (TLR3) agonist, synthetic double-stranded RNA polyriboinosinic polyribocytidylic acid, Poly (I:C) [17]. Poly (I:C) has been known to activate nuclear factor kappa-light-chain-enhancer of activated B cells (NFkB) and subsequently the innate cytokines Interferron-alpha (IFNα) and IFNβ, and Tumor Necrosis Factor-alpha (TNFα), and adaptive TH1 type responses [18,19]. We selected the previously pandemic H1N1 influenza swine strain from 2009 as the target virus and used recombinant hemagglutinin or inactivated virus from this strain as antigens.

## 2. Materials and Methods

### 2.1. Vaccine Preparations

Vitamin A palmitate (Vit A) and alpha-tocopherol (Vit E) (Sigma/Aldrich, St. Louis, MO, USA), were mixed into mustard seed oil (Botanical Innovations, Spooner, WI, USA) to form the oil phase. EGCG, (Sigma/Aldrich)) was mixed in 0.1× PBS and then added to the oil phase in the presence of 2% each *v*/*v* Span80 and Tween80 (Sigma Aldrich) and was homogenized at high speed with a Silverson homogenizer to yield an emulsion. The amount used for each vaccine mouse dose was 30 µg for Vit A, 2 mg Vit E, 240 µg for EGCG, and 35% *v*/*v* for seed oil. Recombinant HA protein (H1N1sw 2009) corresponding to 0.25 µg and 0.025 µg per dose was used as indicated in the immunogenicity studies. For the protective efficacy study, the rHA (Protein Sciences, Meriden, CT, USA) dose was 0.4 µg. The total volume for each Intra-muscular (IM) thigh dose was 100 µL, adjusted with Dulbecco’s phosphate buffered saline (Cat# 21-030-CV). All vaccines were prepared with endotoxin free reagents and in endotoxin free 2.0 mL tubes (Eppendorf biopur safe-lock microcentrifuge tubes).

### 2.2. Animals and Immunizations

All animal studies were approved by the IACUC of the University of Connecticut (Animal Welfare Assurance Number: A3124-01) and the Trudeau Institute before the onset of studies. Female inbred Brag Albino strain c (BALB/c) or outbred CD1 Swiss Albino-derived mice (Charles River Laboratories, Wilmington, MA, USA) (5 per group for immunizations studies and six per group for the protective efficacy study) were immunized once or twice intra-muscularly in the thigh (IM) at a 3 week interval. All immunizations were performed on unanesthetized mice. As a comparison, HA mixed with AddaVax™ (Invivogen; a squalene-based oil-in-water nano-emulsion with a formulation similar to MF59^®^ that has been licensed in Europe for adjuvanted flu vaccines, at 1:1 *v*/*v* for antigen:Addavax), was used as a well-known and licensed systemic adjuvant. Serum was collected 21 days after the first immunization or 10 days after the second immunization. For the measurement of T cell responses in systemic tissues, 10 days after the final immunization, mice were euthanized and spleens (SP) were harvested for the detection of antigen-specific T cells as measured by cytokine production. The mice were maintained at the Association for Assessment and Accreditation of Laboratory Animal Care (AAALAC) approved vivarium of the University of Connecticut, Storrs. The investigators adhered to the “Guide for the Care and Use of Laboratory Animals” prepared by the Committee on Care and Use of Laboratory Animals of the Institute of Laboratory Resources, National Research Council.

### 2.3. Standard Colorimetric ELISA for Antibody Titers

Titration of HA-specific Immunoglobulin G (IgG) was performed on serum from individual mice collected at 3 weeks after the 1st or 10 days after the second immunization. Maxisorp 96-well flat-bottom plates (Nunc, Roskilde, Denmark) were coated overnight at 4 °C with 0.6 μg/mL HA in phosphate-buffered saline pH 7.4 (PBS). The coated wells were blocked for 1 h at room temperature with 300 μL of PBS pH 7.4, 0.1% BSA, and 0.05% Tween-20 with 1% goat serum. The plates were washed with PBS pH 7.4, 0.1% BSA, and 0.05% Tween-20, tapped. Serum samples were initially diluted 1:300 with the dilution buffer (PBS pH 7.4, 1% BSA, 0.05% Tween-20), then transferred into coated-blocked plates in which the samples were serially diluted three-fold with the same buffer. Antigen specific IgG1, IgG2b, IgG2a, and IgA titers were revealed with HRP-conjugated goat anti-mouse IgG1, IgG2b, IgG2a, and IgA (Southern Biotech Associates, Al, USA). Antibody titers are expressed as the logarithm of the enzyme-linked immunosorbent assay titers that give an optical density (OD) higher than the mean plus five times the standard deviation (SD) of the average OD obtained in the pre-immune or naïve sera.

### 2.4. Measurement of Cytokine and Chemokine Responses

For the detection of serum innate responses, sera were collected at 16 hours following the first IM vaccination and analyzed for concentrations of G-CSF, IL-5, MCP-1, MIP1, MIP-1β, IL-6, IP10, KC, MIP-2, RANTES, and TNFα by the Luminex multiplex assay using a Millipore MAGPIX instrument. For the detection of adaptive TH1 (IL-2, IFNγ, granzyme B), TH2 (IL-5, IL-13), TH17 (IL-17A/F), and Treg (IL-10) cytokine responses and all chemokine responses, single-cell suspensions from SP were cultured overnight in 24 well plates at a concentration of 5 × 10^6^ per mL in the presence or absence of 0.5 μg/mL HA from the 2009 H1N1 swine strain. Supernatants were collected and stored at −80 °C. Innate cytokines and chemokines and adaptive cytokines as stated above, were measured by the Multiplex Luminex Assay using an in-house MAGPIX (Millipore, Billerica, MA, USA) instrument and using Millipore kits as per the manufacturer’s protocol.

### 2.5. Measurement of Serum Amyloid P Component by ELISA

Mouse Serum Amyloid P (SAP) protein was measured by endpoint enzyme linked immunosorbent assay (ELISA). The assay was performed using the Mouse Serum Amyloid P (SAP) ELISA Test Kit (Life Diagnostics, Inc., West Chester, PA, USA, Cat# SAP-1) according to the manufacturer’s protocols. Briefly, microtiter wells were coated with a peptide-specific antibody that recognizes different epitopes on mouse SAP. Another peptide-specific antibody is conjugated to Horseradish peroxidase (HRP) and used for detection. Serum samples collected from mice at 16 hours after a single IM vaccination with HA alone, or HA mixed with NIDS, Sq. oil, Poly (I:C) or Imject Alum were diluted 1:20 and 100 µL of each sample were added to the pre-coated wells and incubated with 100 µL of HRP-conjugate for an hour on a micro-plate shaker at 150 rpm at room temperature. 100 µL SAP standards were diluted at the following concentrations, 500, 250, 125, 62.5, 31.25, 15.63, and 7.81 ng/mL and incubated with the HRP-conjugated secondary antibody for an hour on the shaker. Unbound HRP-conjugate were washed away with washing buffer included in the kit. Next, 100 µL of TMB Reagent were added to the wells and the plates were incubated for 20 min at room temperature. Color development was stopped using the stop reagents included in the kit. Using a plate reader, the optical density was measured at 450 nm and the serum SAP concentrations were calculated from the standard curve and expressed as ng per mL serum.

**Isolation of Serum:** Mice were bled through the mandibular vein one day prior to the beginning of the challenge study. In brief, mice were scruffed by the neck. The grip was tight enough so that the cheek was taut, but did not restrict respiration of the mouse. The slight depression in the fur near the middle/rear of the mandible was located. Gently, in an upward motion toward the mandible, an 18 gauge needle penetrated the skin not further than 1 mm. Blood was collected into the BD Microtainer with serum separator tubes. Blood was left at room temperature for 30 min. Tubes containing blood was spun at 10,000 rpm for 10 min. Serum was isolated and placed into a fresh Eppendorf tube and stored at −20 °C.

**Hemagglutination Inhibition Assay:** The Hemagglutination Inhibition assay detects serum antibodies to the viral hemagglutinin by measuring the ability of antibodies to inhibit virus-mediated agglutination of erythrocytes. In brief, chicken red blood cells (RBCs) (Charles River) were washed 3–5 times in PBS and resuspended at a final concentration of 1%. The influenza virus was thawed and adjusted to appropriate hemagglutination units in 0.05 mL of PBS. Serum was initially diluted in PBS 1 to 10 and serially diluted in five-fold increments resulting in dilutions from 1:10 to 1:781,250. Then 0.025 mL of diluted serum was added to the 0.05 mL of the virus and incubated for 30 min at room temperature. After this incubation, 0.05 mL of 1% RBCs was added to every well and incubated for one hour at room temperature. Plates were observed for agglutination, with nonagglutinating cells being defined as a button appearance of cells at the bottom of the well. The Hemagglutination Inhibition Assay titer of the serum samples was determined to be the inverse of the last dilution where cells were not agglutinated.

**Viral Stock Preparation for Intranasal Administration:** The virus stock of the H1N1 2009 swine influenza virus strain and clade was retrieved from the −80 °C freezer and quickly thawed. The EID_50_ stock was serially diluted to a concentration of 1.0 × 10^5^ EID_50_ in PBS without Ca^2+^/Mg^2+^.

**Anesthesia:** Animals were anesthetized using an Isoflurane special circuit system (Surgivet Anesthesia Machine CDS9000, SurgieVet Smiths Meidcal, 5200 Upper Metro Place, Suite 200, Dublin, OH 43017, USA, Tel.: +1-800-258-5361); working setup: 3% Isoflurane in O_2_ at 1.75 L/min O_2_ at 14.7 PSIA flow rate. Animals were subjected to anesthesia prior to viral infection and prior to each IN instillation.

**Viral Infection:** Anesthetized mice were infected intranasally with the LD50 (50,000 EID_50_) of the H1N1 2009 swine Influenza virus in 50 µL PBS without Ca^2+^/Mg^2+^.

**Monitoring:** Following IN instillations, and during the postoperative recovery period, mice were observed for possible respiratory disorders (dyspnoea, partial aspiration, etc.). Mice were observed as they recovered from anesthesia and again one day later for signs of illness and to be sure they were eating and drinking as approved and reviewed by the IACUC committee. Follow up: Survival, weight, and clinical scoring were performed daily starting prior to infection on day 0 (baseline), until death or scheduled study termination, whichever came first. Clinical scoring was performed by a person blinded to the study design and animal’s identity, based on the following scale:

0 = no visible signs of disease;
1 = Slight ruffling of fur;
2 = ruffled fur, reduced mobility;
3 = ruffled fur, reduced mobility, rapid breathing;
4 = ruffled fur, minimal mobility, huddled appearance, rapid and/or laboured breathing;
5 = death/euthanize.

**Unscheduled Euthanasia/Animal Death:** If animals displayed moribund signs (see below), they were humanely euthanized and recorded as dead on the particular day. Mice, once infected, were monitored daily for weight changes and euthanized humanely immediately if they became recumbent, failed to move upon stimulation, exhibited an inability to eat or drink, or if they lost > 30% of their initial body weight as stipulated and approved by the Institutional Animal Care and Use Committee (IACUC).

**Scheduled Euthanasia and Lung Harvest:** Animals from the challenge study were euthanized on day 6 after the viral infection, through intaperitoneal injection of tribromoethanol followed by exsanguination. Lungs were harvested into a 2 mL Sample Tube RB (Qiagen) with 1 mL of PBS without Ca^++^ or Mg^++^. Tissue will be homogenized using a TissueLyser II (Qiagen). Samples were spun down at 12,000 rpm for 10 min at 4 °C. The lung homogenate was then split into two 2.0 mL cryovials and stored at −80 °C until a viral plaque assay was performed to determine the viral titers within each lung sample.

**Viral Load Determination:** Viral titers were determined using a viral foci assay using Nitro Blue Tetrazolium. In brief, Madin-Darby Canine Kidney Epithelial Cells (MDCK) cells were seeded into a 96 well plate and grown to confluence. Media was replaced with Trypsin/Zero-Serum media while viral dilutions were made. Lung homogenate was serially diluted using aerosol barrier tips in a 1:10 initial dilution followed by 1:5 serial dilutions. Trypsin/Zero-Serum media were then removed from the 96 well plate and diluted lung homogenate was added. Plates were spun at 700× *g* for 1.5 h. Sample dilutions were then aseptically aspirated and Trypsin/Zero-Serum media were added to the cells. Plates were incubated for 24 h at 32 °C, 5% CO_2_. After 24 h, plates were aspirated and fixed with acetone for 30 min at −20 °C. Next, acetone was removed and plates were allowed to dry completely. Once dry, plates were washed in staining buffer and then incubated with mouse anti-influenza A-biotin monoclonal antibody for 1 h at 37 °C. After primary antibody incubation, plates were washed with staining buffer and then stained using streptavidin-alkaline phosphatase for 1 h at 21 °C. After the incubation, the plates were washed with staining buffer and developed using nitro-blue tetrazolium and 5-bromo-4-chloro-3′-indolyphosphate (BCIP/NBT) substrate for 0.5 to 1.0 h at 21 °C. Once maximal spot development occurred, the substrate was removed via aspiration and the plates dried at room temperature overnight. Viral foci were counted the following day using an inverted dissecting light microscope with a 4× objective lens.

**Survival of Animals:** Daily recording was performed until scheduled study termination.

**Lung Viral Loads of the Animals:** Both raw data of individual animals and average/mean data were analyzed by appropriate statistical methods as described below.

### 2.6. Statistical Analysis

Results shown are from one representative study with 5 mice per group for the immunogenicity studies. Each study was repeated twice, i.e., a total of 3 independent studies, and very similar results were obtained. For the protective efficacy study, twelve mice per vaccination group were challenged following two IM vaccinations, divided into two subgroups of 6 mice each. Following challenge, subgroups with 6 mice each were monitored for survival and weight loss, and the other subgroups of 6 mice each were sacrificed on day 6 post challenge to measure lung viral loads. The data are presented as mean values from 5 or 6 mice per group ± SEM (error bars). Statistical significance is represented as * *p* ≤ 0.05, ** *p* < 0.01 and *** *p* < 0.001 as shown in the respective figures. Statistical significance was determined by Student’s *t* test, using Microsoft Office software.

## 3. Results

### 3.1. Early Serum Innate Cytokine and Chemokine Responses in Mice Following a Single IM Vaccination

**Figure 1:**
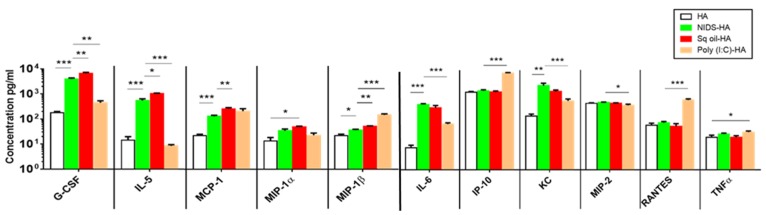
Adjuvant effects of Natural Immune-enhancing Delivery System (NIDS) vs. other adjuvants on early innate pro-inflammatory cytokines and chemokines in BALB/c mice following a single Intra-muscular (IM) vaccination. Comparison of early innate cytokine and chemokine (G-CSF, IL-5, MCP-1, MIP1, MIP-1β, IL-6, IP10, KC, MIP-2, RANTES, and TNFα) responses measured in serum collected 16 h following a single IM vaccination using the Luminex multiplex assay. The data are presented as mean values from five mice per group ± SEM (error bars). Statistical significance is represented as * *p* ≤ 0.05, ** *p* < 0.01 and *** *p* < 0.001. One representative study of three with similar results is shown.

In initial efforts to develop a safer and more efficacious vaccine against pandemic influenza, we measured several serum innate cytokines and chemokines, i.e., G-CSF, IL-5, MCP-1, MIP1, MIP-1β, IL-6, IP10, KC, MIP-2, RANTES, and TNFα. To this end, serum was collected from mice at 16 h after a single IM injection with HA alone or mixed with NIDS, Sq. oil, or Poly (I:C). Serum concentrations of G-CSF, IL-5, IL-6, KC, MCP-1, and MIP-1β were significantly enhanced following IM vaccination with NIDS compared to the antigen alone (Figure 1) (*p* < 2.78 × 10^−8^–*p* < 0.02). Furthermore, vaccination with Sq. oil significantly enhanced the same cytokines and chemokines as NIDS, compared to the antigen alone (*p* < 3.51 × 10^−9^–*p <* 0.01).

Vaccination with Sq. oil resulted in significant increases of G-CSF, IL-5, MCP-1, and MIP-1β (Figure 1) levels compared to vaccination with NIDS (*p* < 0.002–*p* < 0.016). Furthermore, Poly (I:C) induced significantly higher serum IP-10, MIP-1β, and RANTES compared to vaccination with the antigen alone (Figure 1) (*p* < 1.26 × 10^−8^–*p* < 8.96 × 10^−6^), or vaccination with NIDS (*p <* 2.02 × 10^−7^–*p* < 2.95 × 10^−6^). In addition, vaccination with NIDS significantly enhanced G-CSF, IL-5, IL-6, KC, and MIP-2 compared to Poly (I:C) (Figure 1) (*p* < 3.12 × 10^−7^–*p* < 0.033). While TNFα levels showed no significant change in mice vaccinated with NIDS and Sq. oil, vaccination with Poly (I:C) significantly increased TNFα levels compared to the antigen alone (*p* < 0.034) (Figure 1).

These results demonstrate that an IM injection with Sq. oil compared to NIDS resulted in significantly higher concentrations of serum innate IL-5, and the pro-inflammatory chemokines G-CSF, MCP-1, and MIP-1β. Furthermore, an IM injection with Poly (I:C) compared to NIDS induced significantly higher serum innate RANTES, MIP-1, and IP-10. Hence, compared to Sq. oil or Poly (I:C), vaccinations with NIDS resulted in significantly lower select innate pro-inflammatory cytokines or chemokines.

### 3.2. Vaccinations with HA Mixed with NIDS Significantly Enhanced Serum Antibody Responses Compared to IM Vaccinations with HA Alone, or HA Mixed with Sq. Oil or Poly (I:C)

We next determined whether NIDS would exert any adaptive immune enhancing effects when used as an adjuvant compared to other licensed or in development adjuvants. Groups of mice were vaccinated twice IM with HA alone, or HA mixed with NIDS, Sq. oil, or Poly (I:C).

**Figure 2:**
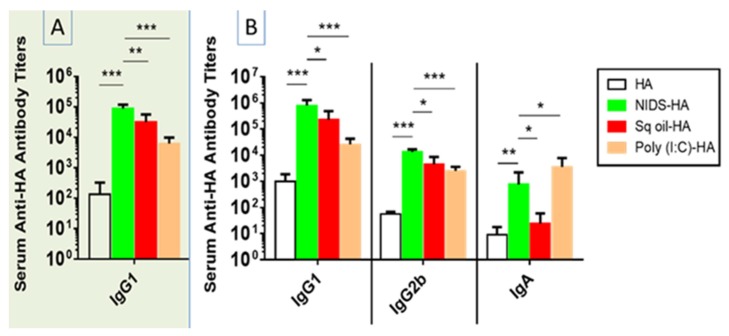
Effects of NIDS vs. other adjuvants on antibody responses against the Hemagglutinin HA antigen following one or two intra-muscular (IM) vaccinations in BALB/c mice. (**A**) Anti-HA IgG1 end point antibody titers measured by ELISA in sera collected at three weeks post 1st intra-muscular (IM) vaccination; (**B**) Anti-HA IgG1, IgG2b, and IgA end point antibody titers following two IM vaccinations collected at ten days post 2nd vaccination measured by ELISA. The data are presented as mean values from five mice per group ± SEM (error bars). Statistical significance is represented as * *p* ≤ 0.05, ** *p* < 0.01 and *** *p* < 0.001. One representative study of three with similar results is shown.

Vaccination with HA mixed with NIDS significantly enhanced serum anti-HA IgG1 responses at three weeks following a single IM vaccination compared to vaccination with HA alone (*p* < 0.001), or HA mixed with Sq. oil (*p* < 0.01) or Poly (I:C) (*p* < 0.001) (Figure 2A). Similarly, significantly enhanced serum anti-HA IgG1 responses were observed following two IM vaccinations with HA mixed with NIDS compared to HA alone (*p* < 0.001) and HA mixed with Sq. oil (*p* < 0.05) or Poly (I:C) (*p* < 0.001) (Figure 2B). In addition, vaccination with HA mixed with NIDS resulted in significantly higher serum anti-HA IgG2b responses compared to HA alone (*p* < 0.001), or HA mixed with Sq. oil (*p* < 0.05) and Poly (I:C) (*p* < 0.001) (Figure 2B). Interestingly, vaccinations with HA mixed with NIDS significantly enhanced serum anti-HA IgA responses compared to HA alone (*p* < 0.01) and HA mixed with Sq. oil (*p* < 0.05), but not compared to HA mixed with Poly (I:C) (Figure 2B). Of note, we also tested the adjuvant activity of Alum (Invivogen, used at 33% *v*/*v*), but the responses were poor and comparable to no adjuvant results (data not shown here, but shown in the Supplemental Data). These data demonstrate that the NIDS adjuvant was superior for the induction of serum antibody responses compared to the other adjuvants.

### 3.3. Vaccinations with NIDS Significantly Enhanced Adaptive TH1 and TH2 Cytokine Responses Compared to Vaccinations with No Adjuvant, or with Other Adjuvants

**Figure 3:**
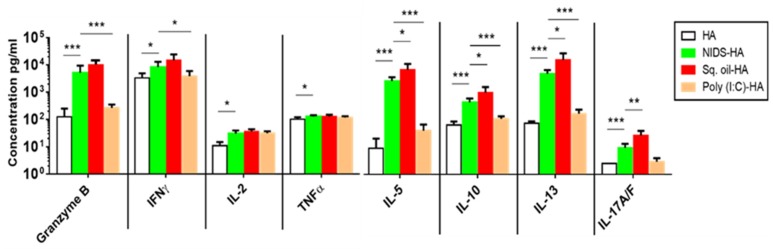
Cellular responses in spleen following two intramuscular vaccinations with NIDS compared to HA alone or other adjuvants. Groups of five mice each were vaccinated as described under the Figure 2 legend. Single cell suspensions were prepared from spleens. Adaptive T Helper 1 (TH1), TH2, TH17, and Treg cytokine responses were measured in splenocyte (5 × 10^6^) supernatants following activation for 72 h with HA. Assays used were ELISA (IL-17) or multiplex Luminex (all other cytokines). Granzyme B, IFNγ, TNFα, IL-2, IL-5, IL-13, IL-10, and IL-17A/F. The data are presented as mean values from five mice per group ± SEM (error bars). Statistical significance is represented as * *p* ≤ 0.05, ** *p* < 0.01 and *** *p* < 0.001. One representative study of three with similar results is shown.

To support the findings on the adaptive antibody response, we next examined the adaptive cellular responses in spleens following two IM vaccinations. Vaccinations with HA mixed with NIDS compared to vaccinations with HA alone, resulted in significantly enhanced TH1 and TH2 cytokines (Figure 3). Vaccinations with HA mixed with NIDS resulted in significantly enhanced Granzyme B compared to HA alone (*p* < 0.001) or HA mixed with Poly (I:C) (*p* < 0.001) (Figure 3). Furthermore, vaccinations with NIDS induced significantly higher TH1 (IFNγ, TNFα, IL-2) as well as TH2 (IL-5, IL-13) compared to vaccinations with HA alone. In addition to enhancing both TH1 and TH2 responses, vaccinations with NIDS also induced a significantly higher regulatory T cell (Treg) response (IL-10) compared to HA alone (Figure 3). Compared to Poly (I:C), NIDS also induced significantly higher IFNγ, IL-5, IL-13, and IL-10 (Figure 3). In contrast, vaccination with Sq. oil induced significantly higher TH2 (IL-5 and IL-13) and Treg (IL-10) cytokines compared to NIDS (Figure 3). Interestingly, vaccination with NIDS resulted in significantly lower IL-17A/F compared to Sq. oil (Figure 3), and in two other independent studies, vaccinations with HA mixed with NIDS did not induce significantly higher splenic IL-17 responses compared to vaccinations with HA alone (data not shown). Thus, vaccinations with NIDS induced balanced TH1 and TH2 responses, whereas vaccinations with Sq. oil induced significantly higher TH2 and TH17 responses compared to vaccinations with NIDS.

### 3.4. Vaccinations with HA Mixed with NIDS Compared to HA Alone Significantly Enhanced Serum HI Titers and Significantly Reduced Lung Viral Pfu Titers and Clinical Scores following Intra-Nasal Challenge with Live Influenza Virus

Based on the promising results of the innate and adaptive antibody responses from the immunogenicity studies, we further examined the protective efficacy of NIDS in BALB/c mice. The protective efficacy study was performed in a single blind fashion, in that the personnel involved with this study were withheld information on the adjuvant used in each vaccination group. The mice were vaccinated twice IM with recombinant HA antigen or inactivated A/Cal/04/09 influenza virus alone or mixed with NIDS or Sq. oil. In addition to measuring the serum anti-HA neutralizing activity by the Hemagglutination Inhibition (HI) assay following two IM vaccinations, clinical signs and lung virus pfu titers were also measured.

**Figure 4:**
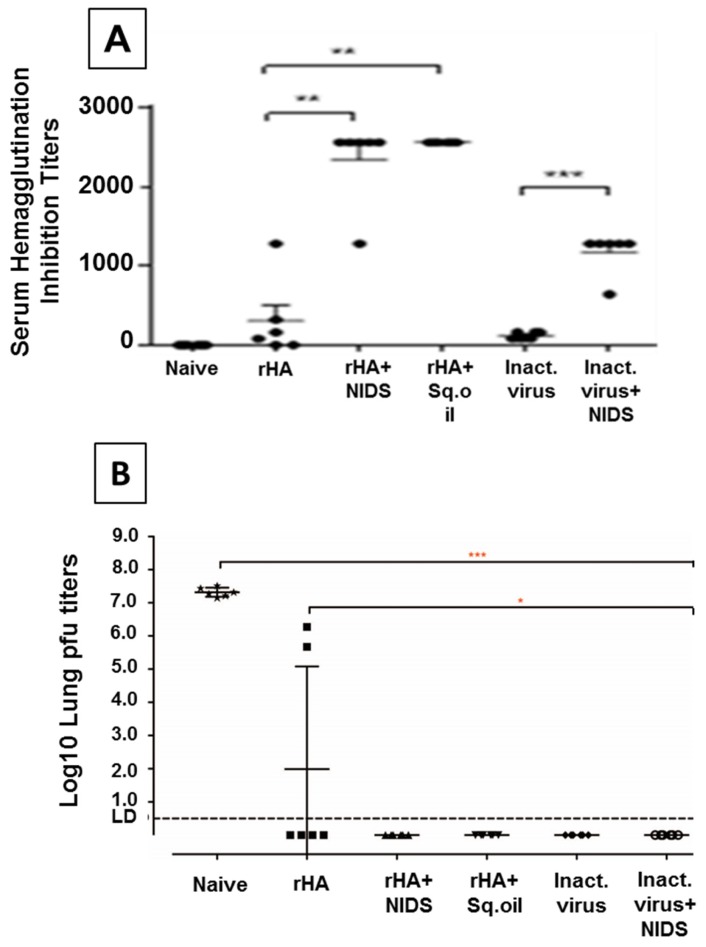

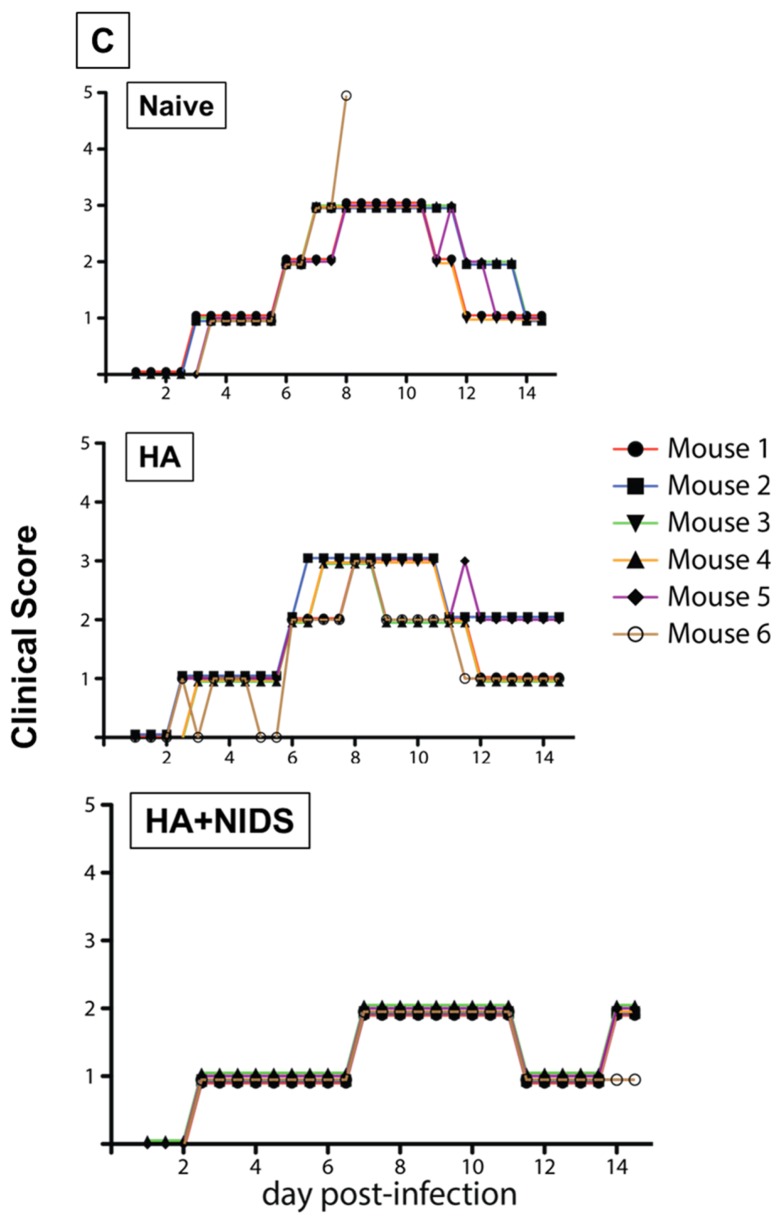
Protective efficacy of NIDS. Mice were vaccinated three weeks apart, twice intramuscularly (IM). Groups of six mice per group were vaccinated with HA alone, HA mixed with NIDS, or HA mixed with Sq. oil. Mice were also similarly vaccinated twice IM with inactivated virus alone, or with inactivated virus mixed with NIDS. (**A**) Hemagglutination inhibition titers were measured in sera collected 2 weeks post 2nd vaccination; (**B**) Mice were challenged 3 weeks post 2nd vaccination intra-nasally with live influenza and Lung pfu titers were measured; (**C**) Clinical scores of mice following vaccinations and intranasal challenge with live influenza virus. The data in (**A**,**B**) are presented as mean values from six mice per group ± SEM (error bars). Each dot or line represents an individual mouse within a specific vaccination group. Differences with statistical significance between groups are denoted with lines with one or more asterisk, represented as * *p* ≤ 0.05, ** *p* < 0.01 and *** *p* < 0.001.

Following two systemic vaccinations with HA mixed with NIDS, significantly higher HI titers were measured compared to vaccinations with HA alone (*p* < 0.01), but were not compared with vaccinations with HA mixed with Sq. oil (Figure 4A). Moreover, inactivated virus mixed with NIDS also induced significantly enhanced HI titers compared to vaccination with inactivated virus alone (*p* < 0.001) (Figure 4A). Intra-nasal (IN) challenge of unvaccinated/naïve mice resulted in significantly higher lung pfu titers compared to all other groups (*p* < 0.001). Importantly, vaccinations with HA alone resulted in significantly higher lung pfu titers compared to vaccinations with HA mixed with NIDS (*p* < 0.05) (Figure 4B).

As a further measure of the protective efficacy of NIDS, we also compared the clinical scores of the mice, vaccinated with HA mixed with NIDS compared to vaccinations with HA alone and no vaccinations (naïve). We found significantly higher clinical scores in unvaccinated mice as well as in mice vaccinated with HA alone, compared to mice vaccinated with HA mixed with NIDS (Figure 4C).

### 3.5. Vaccinations with NIDS Had a Higher Safety Profile as Measured by Serum Amyloid P Component and Total Serum IgE

**Figure 5:**
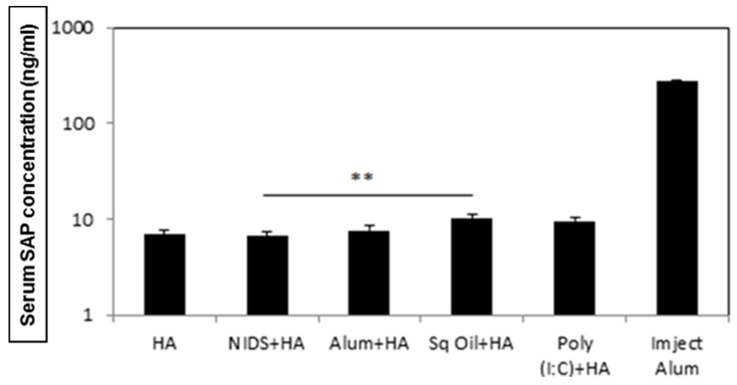
Significantly enhanced Serum Amyloid P component (SAP) concentrations in mice at 16 h following a single Intra-muscular vaccinations with Sq. oil + HA compared to NIDS + HA or HA alone. The data are presented as mean values from four mice per group ± SEM (error bars). Imject Alum was used as the positive control for the assay. Statistical significance is represented as ** *p* < 0.01.

SAP concentrations, used as a measure of liver induced toxicity, were significantly enhanced in mice vaccinated once IM with HA mixed with Sq. oil (*p* < 0.01) compared to the vaccinations with HA alone or with HA mixed with NIDS (Figure 5). Vaccination with NIDS mixed with HA did not enhance serum SAP concentrations compared to vaccination with HA lone. As a control, three IM vaccinations with HA adsorbed to Imject Alum induced significantly higher SAP concentrations compared to all other vaccination groups. Thus, vaccinations with the NIDS adjuvant proved safer than vaccinations with Sq. oil.

**Figure 6:**
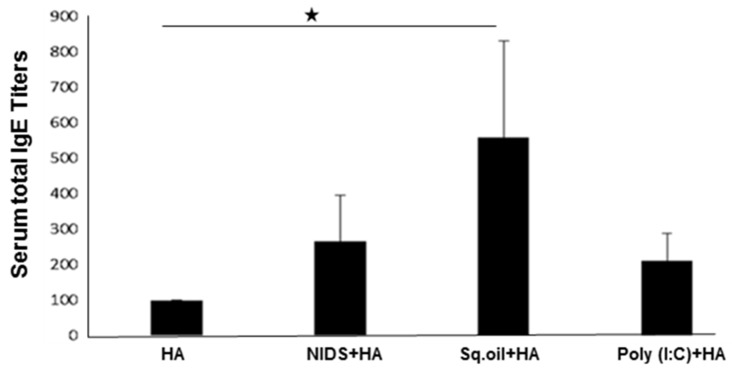
Total serum IgE concentrations following two Intra-muscular vaccinations with HA alone, HA mixed with NIDS, HA mixed with Sq. oil, and HA mixed with Poly (I:C). The data are presented as mean values from five mice per group ± SEM (error bars). Statistical significance is presented as single asterisk denoting *p* < 0.05.

While antigen-specific IgE antibodies are known to be important for protection against parasitic pathogens, sustained high levels of IgE may exacerbate allergic reactions. Therefore, as a further measure of safety, we next measured total serum IgE levels in mice vaccinated with HA alone compared to those vaccinated with HA mixed with NIDS, Sq. oil, or Poly (I:C). We found that two IM vaccinations with Sq. oil induced significantly higher serum IgE titers compared to vaccinations with HA alone (*p* < 0.05; Figure 6). In contrast, vaccinations with HA mixed with NIDS or Poly (I:C) did not induce significantly higher serum IgE levels compared to vaccinations with HA alone (Figure 5). These data demonstrate that vaccinations with NIDS were safer than vaccinations with Sq. oil as measured by the SAP and total serum IgE levels.

### 3.6. NIDS Adjuvant Demonstrated Strong Dose Sparing Effects on Adaptive Serum Antibody Responses

**Figure 7:**
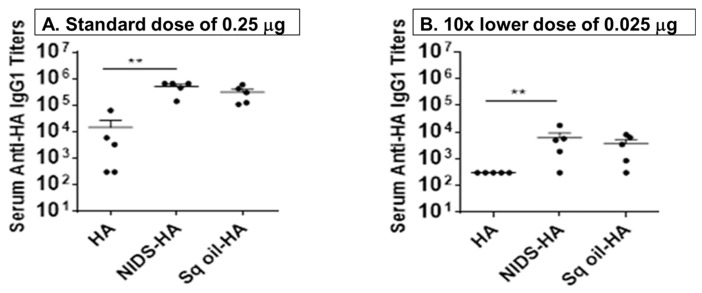
Dose sparing effects of NIDS compared to other adjuvants on antibody responses. (**A**) Serum anti-HA IgG1 titers following two systemic vaccinations with the standard (0.25 µg) dose of HA antigen measured by ELISA; (**B**) Serum anti-HA IgG1 titers following two systemic vaccinations with 10× lower (0.025 µg) dose of HA antigen measured by ELISA. The data are presented as mean values from five mice per group ± SEM (error bars). Each dot represents an individual mouse within a specific vaccination group. Statistical significance is represented as ** *p* < 0.01.

We further evaluated the dose sparing effects of the HA antigen in BALB/c mice using the standard dose (i.e., the relatively low dose of 0.25 µg HA used in all the studies) and 10× lower dose (0.025 µg) following two IM vaccinations with HA alone or HA mixed with NIDS, or Sq. oil. Sera collected at ten days post 2nd vaccination were used to measure antibody responses using ELISA. Vaccination with HA mixed with NIDS resulted in significantly higher serum anti-HA IgG1 responses compared to vaccinations with HA alone with both the standard dose (Figure 7A) and 10× lower dose (Figure 7B). Additionally, either dose of HA (standard or 10× lower) adsorbed to Alum resulted in no change in serum anti-HA IgG1 responses compared to HA alone (data not shown).

### 3.7. The Adjuvant Effect of NIDS Is Intact on Antibody Responses in Outbred (CD1) Mice

**Figure 8:**
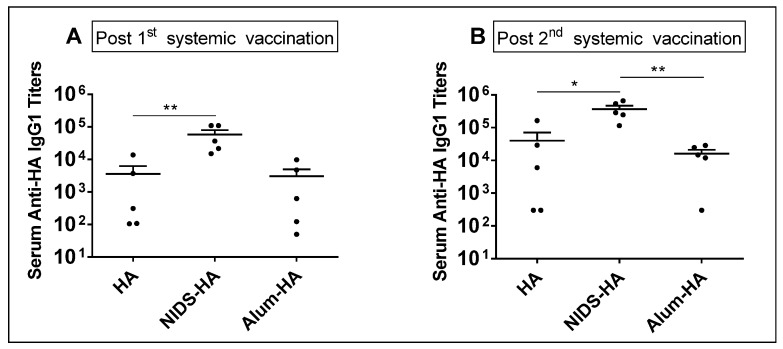
Adjuvant effect of NIDS in the outbred CD1 mice on serum antigen specific antibody responses following IM vaccinations. (**A**) Anti-HA IgG1 antibody titers in sera collected at 3 weeks following a single IM vaccination; (**B**) Anti-HA IgG1 antibody titers in sera collected at 10 days post 2nd IM vaccination. Vaccination with NIDS induced significantly higher IgG1 responses compared to HA alone as well as HA adsorbed to Alum. The data are presented as mean values from five mice per group ± SEM (error bars). Each dot represents an individual mouse within a specific vaccination group. Statistical significance is represented as * *p* ≤ 0.05, ** *p* < 0.01.

We further evaluated the adjuvant effect of NIDS on antibody responses in the outbred CD1 mice following one or two IM vaccinations with HA alone or HA mixed with NIDS, or adsorbed to Alum. Vaccination with HA mixed with NIDS induced significantly enhanced serum anti-HA IgG1 responses following a single IM vaccination compared to HA alone (Figure 8A). Additionally, two IM vaccinations with HA mixed with NIDS resulted in significantly higher serum anti-HA IgG1 responses compared to HA alone (*p* < 0.05) or HA adsorbed to Alum (*p* < 0.01) (Figure 8B). Thus, the adjuvant effect of NIDS was intact in the outbred CD1 mice, which better represent the outbred human population.

## 4. Discussion

This is the first study demonstrating that a natural vaccine adjuvant, i.e., NIDS, comprising a combination of nutritive compounds, i.e., Vit A, Vit E, and a catechin (EGCG) in a vegetable oil in water emulsion conferred complete protective efficacy against intra-nasally induced infection with live influenza virus. The immunomodulatory properties of each of the components of NIDS have been discussed extensively in two recent reviews [10,11]. Moreover, the adjuvant effect of the NIDS has been demonstrated in two recent publications, in which NIDS was shown to enhance adaptive CD4+ TH, CD8+ IFNγ+, and B cell antibody responses [8,9]. These studies demonstrated that the combinations of Vit A or Vit E and EGCG formulated as an oil in water emulsion using a vegetable oil, synergistically enhanced adaptive immune responses, compared to the vitamins alone in oil or EGCG alone in oil. While the detailed mechanism of action of this novel adjuvant remains to be elucidated, our studies thus far suggest that innate IL-15 responses may be required [9]. In addition, due to the high anti-oxidant activity of the NIDS, i.e., Vit A, Vit E, and EGCG, daily oral treatment of mice with NIDS resulted in enhanced expression of super oxide dismutase 1 (SOD1), which converts superoxide radicals to free oxygen [9]. In addition, our preliminary data indicate that vaccinations with NIDS enhanced mitochondrial health as well as reduced apoptosis in the draining lymph nodes within hours after a single IM injection (data not shown). Considering the lower innate pro-inflammatory responses shown in this study, if the above preliminary data can be established, taken together, these data could suggest that the NIDS may exert its adjuvant effect through induction of improved mitochondrial health, and reduced apoptosis as a result of reduced pro-inflammatory responses, as opposed to most pro-inflammatory vaccine adjuvants and delivery systems which result in more rapid proliferation and cell death.

It is important to note that the NIDS can be prepared as an all natural vaccine adjuvant, and all the components are available as Good Manufacturing Practice (GMP) grade material. Moreover, since it can be prepared as a nano-emulsion with droplet sizes of 200 nm, it can be readily sterile filtered. Thus, this novel adjuvant has immediate industrial applications to be used against existing or emerging pathogens.

In these studies we established that NIDS enhanced some systemic innate responses, but not others that may be related to adverse clinical events. Specifically, innate G-CSF, IL-5, MCP-1, MIP-1α, and MIP-1β responses induced following injection with NIDS were significantly lower than the responses induced by Sq. oil. Moreover, innate IP-10, RNATES, and TNFα responses induced following injection with NIDS were significantly lower compared to the responses induced by Poly (I:C).

As safety biomarkers, besides early serum innate inflammatory responses, we also measured SAP and total IgE. Normally, serum levels of APR go back to normal levels 1–2 days after the inflammatory response. However chronic inflammation can cause APR levels to remain abnormal which can be detrimental and can cause complications and toxicity issues [12]. APR levels can be measured in mice to determine how vaccinations with different adjuvants trigger an inflammatory response as well as the strength and duration of this response. Therefore, in this study, we chose to measure the APR Serum Amyloid P component (SAP). SAP serum levels increase measurably upon an inflammatory reaction. In mice, SAP has a normal serum level of 20–150 ng/mL, depending on the strain, and acts as an opsonin by binding to apoptotic cells and antigens and promoting phagocytosis. However, in a chronic inflammation, SAP can be toxic and may cause amyloidosis [13,14].

There are several lines of evidence that strongly suggest that influenza infection exacerbates allergic reactions in sensitized individuals in which IgE may play an important role [20,21]. Infection with pandemic influenza strains cause morbidity and mortality preferentially in children and young adults [22]. This is due to a strong pro-inflammatory response in the lung. Hence it is conceivable that pre-sensitization of the general population to a TH2 type response and IgE, may further exacerbate the respiratory distress caused by infection with pandemic or seasonal influenza viruses. Support for such a hypothesis is also drawn from the respiratory syncytial virus infections and vaccines, in which case in a clinical trial performed decades ago, vaccinations with inactivated Respiratory Syncytial Virus (RSV) adsorbed to Alum exacerbated respiratory distress in vaccinated infants who were subsequently infected with RSV, two of whom died. Hence, it may arguably be safest to vaccinate against pandemic or seasonal influenza with vaccines that do not cause overt TH2 and IgE responses. The SAP and IgE results, together with the induction of low to no innate (shown previously) and adaptive (shown here) IL-17 responses, strongly argue for a significantly higher safety profile of NIDS. Moreover, the data hold promise to use these biomarkers for vaccine safety that may be translated across higher mammalian species and humans.

In a recent publication, we reported that local, site of injection, pro-inflammatory cytokines and chemokines were significantly decreased in a murine air pouch model following the injection of NIDS compared to Poly (I:C) or imject Alum [9]. Moreover, IM vaccinations with NIDS induced lower early serum innate IP-10 responses compared to Poly (I:C), whereas vaccinations with NIDS induced significantly higher IL-5, KC, and G-CSF compared to vaccinations with no adjuvant. These data are in agreement with the data shown in the present study. Moreover, the decreased innate and adaptive IL-17 responses in the previous publication and the current study are also in agreement.

Importantly, the NIDS significantly enhanced serum IgG1, IgG2b, and IgA antibody responses as well as adaptive splenic TH1 and TH2 but not TH17 responses compared to vaccination with other adjuvants such as Sq. oil or Alum. The Alum data from the BALB/c mice (shown in the Supplemental Data) demonstrated a lack of enhanced adaptive responses, which was similar to the data shown in the CD1 mice. In addition, vaccinations with HA mixed with NIDS induced significantly higher serum HI titers compared to vaccinations with no adjuvant, regardless of whether the antigen was purified recombinant HA or inactivated influenza virus. Most importantly, vaccinations with NIDS followed by IN challenge with live influenza virus, induced significant protection compared to vaccination with HA alone, as demonstrated by significantly lower lung pfu virus titers and clinical scores. Of note, the adjuvant effect of NIDS, was intact in the outbred CD1 mice, significantly increasing the potential of NIDS exerting its adjuvant effect in the outbred human population.

Studies have shown that higher doses of some pre-pandemic influenza vaccines were required to achieve 50% protection in healthy adults [23,24,25,26,27,28,29]. Also, at least two doses of pre-pandemic vaccinations were required to induce protective responses as opposed to a single vaccination with seasonal influenza vaccines (ibid). Moreover, seasonal influenza vaccinations are not as effective in the elderly and infant populations as they are in individuals aged 18–50. To overcome these problems immune-enhancing adjuvants and delivery systems that are safe, effective, and practical are sought, so that either fewer number of vaccinations or lower doses of influenza antigens can induce enhanced protective efficacy compared to using the antigen alone. It is conceivable that a similar requirement for the dose-sparing property of adjuvants and delivery systems will be needed with regards to other pathogens. In this regard, the strong dose-sparing property of the NIDS in this study was of significant importance. It is noteworthy that in most murine influenza vaccine studies, doses of recombinant HA vary between 0.5–1.0 µg. However, in the current study we used the dose of 0.25 µg which was lowered 10 fold to 0.025 µg, to demonstrate the strong dose-sparing effect of NIDS when mixed together with recombinant HA.

Although TH17 responses have been suggested to play a role in the clearance of infection of certain pathogens, their role in protective efficacy following vaccinations may be less clear. In addition, TH17 responses have been implicated in certain autoimmune diseases [30,31,32,33,34,35].Whether IL-17 innate and adaptive responses following vaccinations are required for protection against influenza infection is currently unknown. A recent study using influenza induced pneumococcal infection, suggested a role of TH17 responses in the protection against middle ear infection following mucosal vaccinations with Pneumococcal surface protein A and Cholera toxin B subunit as adjuvants [36]. The present study demonstrates that despite the induction of low to no innate and adaptive IL17 responses, following vaccinations with NIDS mixed with HA, the protective efficacy against IN challenge of mice was intact.

The influenza virus gains access to the host through mucosal membranes of the upper respiratory tract. Whether long-term mucosal memory immune responses are required for protection against mucosal pathogens has been subject to debate [37]. Factors such as where the pathogen enters vs. where it exerts its pathogenicity, as is the case with, e.g., the polio virus, play an important role on whether local mucosal responses are required for protection. Nonetheless, as long-term immunological memory in the local mucosa may be required for protection against mucosal pathogens [38,39,40], future studies with NIDS will seek to address this issue. In this regard, it is noteworthy that our unpublished data suggest that IN priming followed by IM boosting vaccinations with NIDS mixed with HA compared to IM vaccinations with HA alone conferred significantly higher protection against subsequent IN infection with live influenza.

## Supplementary Materials

The following are available online at http://www.mdpi.com/2072-6643/9/5/516/s1.
