# Supplementary material for: Comparative Safety and Efficacy Profile of a Novel Oil in Water Vaccine Adjuvant Comprising Vitamins A and E and a Catechin in Protective Anti-Influenza Immunity
Source: Nutrients. 2017 May 21;9(5):516. doi: 10.3390/nu9050516 (PMC5452246; doi:10.3390/nu9050516)
Supplement: Supplementary file 1 [file nutrients-09-00516-s001.pdf]

Supplemental Data for MDPI Nutrients

Manuscript ID: nutrients-182470

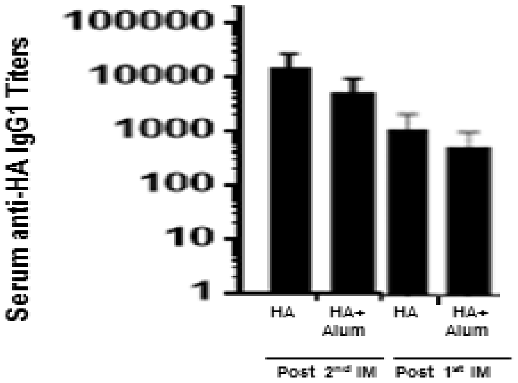

Supplemental Figure. Serum anti-HA IgG1 titers following one or two IM vaccinations in BALB/c mice. Groups of 5 female BALB/c mice were vaccinated intra-muscularly once or twice, at a 3 weeks interval, with recombinant HA alone or adsorbed to aluminum hydroxide (Alum). Sera Were collected at 3 weeks after the first and ten days after the second vaccination And anti-HA specific IgG1 titers were measured by ELISA. This is one representative study of two, showing no significant differences between antibody responses in mice vaccinated with Ha lone or adsorbed to Alum after one or two IM vaccinations.
